# Alternative Protein Secretion in the Malaria Parasite *Plasmodium falciparum*

**DOI:** 10.1371/journal.pone.0125191

**Published:** 2015-04-24

**Authors:** Thuvaraka Thavayogarajah, Preetish Gangopadhyay, Stefan Rahlfs, Katja Becker, Klaus Lingelbach, Jude M. Przyborski, Anthony A. Holder

**Affiliations:** 1 Division of Parasitology, Faculty of Biology, Philipps-University Marburg, Karl-von-Frisch Straße 8, 35043 Marburg, Germany; 2 Biochemistry and Molecular Biology, Interdisciplinary Research Center, Justus Liebig University Giessen, 35392 Giessen, Germany; 3 The Francis Crick Institute Mill Hill Laboratory, The Ridgeway, Mill Hill, London NW7 1AA, United Kingdom; Universidade Federal de Minas Gerais, BRAZIL

## Abstract

*Plasmodium falciparum* invades human red blood cells, residing in a parasitophorous vacuole (PV), with a parasitophorous vacuole membrane (PVM) separating the PV from the host cell cytoplasm. Here we have investigated the role of *N*-myristoylation and two other N-terminal motifs, a cysteine potential S-palmitoylation site and a stretch of basic residues, as the driving force for protein targeting to the parasite plasma membrane (PPM) and subsequent translocation across this membrane. *Plasmodium falciparum* adenylate kinase 2 (*Pf* AK2) contains these three motifs, and was previously proposed to be targeted beyond the parasite to the PVM, despite the absence of a signal peptide for entry into the classical secretory pathway. Biochemical and microscopy analyses of *Pf*AK2 variants tagged with green fluorescent protein (GFP) showed that these three motifs are involved in targeting the protein to the PPM and translocation across the PPM to the PV. It was shown that the N-terminal 37 amino acids of *Pf*AK2 alone are sufficient to target and translocate GFP across the PPM. As a control we examined the N-myristoylated *P*. *falciparum* ADP-ribosylation factor 1 (*Pf*ARF1). *Pf*ARF1 was found to co-localise with a Golgi marker. To determine whether or not the putative palmitoylation and the cluster of lysine residues from the N-terminus of *Pf*AK2 would modulate the subcellular localization of *Pf*ARF1, a chimeric fusion protein containing the N-terminus of *Pf*ARF1 and the two additional *Pf*AK2 motifs was analysed. This chimeric protein was targeted to the PPM, but not translocated across the membrane into the PV, indicating that other features of the N-terminus of *Pf*AK2 also play a role in the secretion process.

## Introduction

*Plasmodium falciparum*, the causative agent of the most severe form of human malaria, is a eukaryotic cell that infects a red blood cell (RBC), which is then extensively modified by proteins exported by the parasite. The parasite develops within a parasitophorous vacuole (PV) delineated by the parasitophorous vacuole membrane (PVM), between the PV and the RBC cytoplasm. The existence of novel pathways to transport proteins to the surface of the parasite cell and beyond has been proposed. Several studies employing reporter constructs support the existence of one or more mechanisms that bypass the canonical N-terminal signal peptide-mediated secretory pathway [1, 2]. In general terms, such secreted proteins are characterised by the absence of a canonical signal sequence or a recessed N-terminal hydrophobic segment [3]. Neither the molecular determinants nor the exact pathways responsible for non-canonical secretion have been identified. The existence of the PV requires additional protein sorting signals and mechanisms to direct export to specific destinations within the PVM, the RBC cytoplasm or the RBC plasma membrane. The discovery of the *Plasmodium* export element (PEXEL)/host targeting (HT) signal—a short amino acid motif—in exported proteins led to the *in silico* identification of approximately 250 proteins predicted to be exported to the host cell (the malarial 'exportome') [4, 5]. Recently, additional exported proteins that do not contain a PEXEL/HT motif were described [6].

*N*-myristoylation is predominantly a co-translational modification of proteins found in all eukaryotic cells. A 14-carbon saturated fatty acid (myristate) is covalently attached *via* an amide bond to the N-terminal glycine residue of the target protein by *N*-myristoyltransferase (NMT). Using prediction algorithms, several proteins encoded in the *P*. *falciparum* genome are putative NMT substrates. Individual proteins have been shown experimentally to be *N*-myristoylated, including adenylate kinase-2 (AK2) [7], ADP-ribosylation factor-1 (ARF1) [8, 9], calcium dependent protein kinase-1 (CDPK1) [10], 45 kDa glideosome associated protein (GAP45) [8], Golgi re-assembly stacking protein 1 (GRASP1) [11], and Rab5b [12]. Recently the myristome of the infected RBC was examined experimentally [13], validating many of the known and predicted NMT substrates.

The myristoyl moiety mediates a reversible binding of the protein to a membrane, for example the plasma membrane or intracellular membranes in eukaryotic cells [14]. It has also been observed that *N*-myristoylation may lead to secretion of the respective protein [15]. For two *N*-myristoylated proteins of parasitic protozoa—*Pf*CDPK1 [10] and the *Leishmania* HASPB [16]—it has been proposed that *N*-myristoylation leads to protein targeting to the plasma membrane and subsequent translocation of the protein across the membrane to the outer surface of the cell. In the *P*. *falciparum* system, *Pf*AK2, which in other organisms plays a role in energy-dependent nucleotide signalling processes [17], was shown to locate to the periphery of the parasite and was proposed to be exported to the PVM [18], suggesting that *N*-myristoylation may play a role in the export of this protein.

These earlier studies prompted us to investigate in detail the subcellular localization of *Pf*AK2 and the elements involved in its targeting. In addition to *N*-myristoylation, two other sequence features at the N-terminus of *Pf*AK2—a putative palmitoylation site and a row of basic amino acid residues—were considered likely to be important. As a control we used *Pf*ARF1, which is a small GTP binding protein involved in vesicular biogenesis and trafficking processes of the secretory pathway in other organisms [19]. Although these proteins differ in their function, both are substrates of *Pf*NMT and therefore appropriate candidates for studying the importance of *N*-myristoylation in subcellular targeting. We used GFP as a fluorescent and antigenic marker to tag the proteins, allowing them to be detected by fluorescence microscopy and western blotting. Their location in different compartments within the infected RBC was examined in detail using streptolysin O and saponin to differentially solubilise the RBC plasma membrane and the PVM, together with accessibility to proteinase K to confirm their membrane topology. Finally, the role of sequence features at the N-terminus of the protein was examined by genetically manipulating the coding sequence and examining the effect on protein location.

## Results

### Fluorescence microscopy and biochemical analyses of PfAK2/GFP suggest a subcellular location outside the parasite in the PV

It was shown previously that GFP-tagged *Pf*AK2 (*Pf*AK2/GFP) was located at the periphery of the parasite in a membrane-bound form, whereas *Pf*AK2^G2A^/GFP was soluble within the cytoplasm of the parasite [18]. To extend these earlier observations, we used live fluorescence imaging and biochemical analyses. The subcellular location of each of the fusion proteins was examined following fractionation of the infected cells; streptolysin O (SLO) was used to lyse the RBC plasma membrane whilst preserving the PVM intact and saponin treatment was used to lyse both the RBC membrane and the PVM. Sensitivity of the fusion protein to Proteinase K digestion was used to confirm the membrane topology of the protein. The pellet fraction after each of the treatments was divided into two aliquots containing equal cell numbers, then one of the aliquots was treated with Proteinase K and the other one was left untreated. The soluble serine rich protein (SERP), which is located in the PV, was used as a control to demonstrate that the PVM remained intact after SLO lysis. *Pf*aldolase, a soluble parasite cytoplasmic protein, was used as a control to show that the parasite remained intact after either SLO or saponin treatment as described [20, 21].

**Fig 1:**
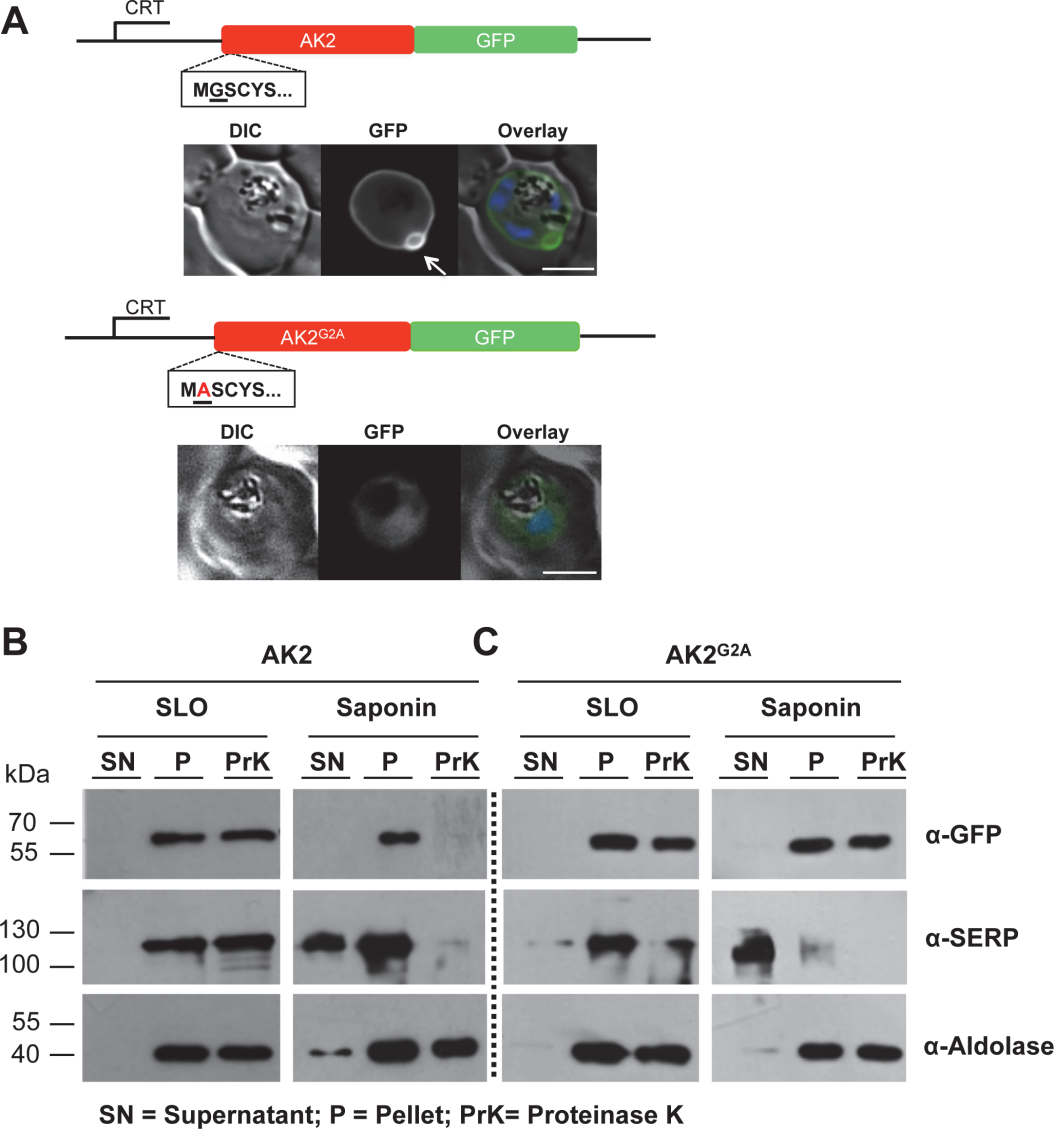
Subcellular location of *Pf*AK2/GFP and the non-myristoylated G2A variant, as determined by live fluorescence microscopy and cell fractionation using SLO and saponin lysis together with a protease protection assay. (A) The AK2/GFP and the AK2^G2A^/GFP fusion proteins were expressed using the CRT promoter from an episomal plasmid (constructs indicated above the images). AK2/GFP is located at the periphery of the intracellular parasite as judged by epifluorescence microscopy, and is associated with one or two protuberances towards the host cell cytoplasm present on each parasite (indicated by white arrow). In contrast, the AK2^G2A^/GFP chimera is located within the parasite cytosol. The infected cell was visualised by differential interference contrast (DIC), intrinsic fluorescence of the GFP identified the location of the AK2/GFP fusion protein, and parasite nuclei were detected by Hoechst staining. Overlay: green (GFP), blue (DNA). Scale bar—3 μm. (B) The *Pf*AK2/GFP transgenic parasite and (C) the G2A variant parasite were both subjected to SLO and saponin lysis, separated into soluble supernatant (SN) and pellet (P) fractions, and then part of the pellet fraction was treated with Proteinase K (PrK). Western blot analysis with equal cell equivalents was performed using anti-GFP, anti-SERP (a soluble PV protein), and anti-aldolase (a parasite cytoplasm protein) antibodies. Size markers are in kDa. The absence of the protein band corresponding to the *Pf*AK2/GFP fusion protein in the saponin pellet fraction treated with Proteinase K indicates secretion of the protein beyond the PPM.

As found previously, by microscopy *Pf*AK2/GFP was at the parasite periphery with one or two distinct protuberances apparently extending away from the parasite into the host cell cytoplasm (indicated with an arrow in Fig 1A). Both the *Pf*AK2/GFP and *Pf*AK2^G2A^/GFP transgenic parasite lines contained a polypeptide of approximately 60 kDa detectable by an anti-GFP antibody in the cell pellet fraction obtained after SLO treatment. This band corresponds to the expected size of the fusion protein consisting of full-length *Pf*AK2 (~ 32.5 kDa) and GFP (~27 kDa) (Fig 1B and 1C). After saponin and Proteinase K treatment of the cellular fraction obtained from *Pf*AK2/GFP parasite-infected RBC, the band of ~ 60 kDa was no longer present. The sedimentation of *Pf*AK2/GFP and its susceptibility to the protease indicated that (i) the protein was membrane-bound, and (ii) exposed to the vacuolar space. In contrast, the *Pf*AK2^G2A^/GFP fusion protein was protected from protease cleavage following either SLO or saponin treatment. This result is consistent with the morphological data indicating that the *Pf*AK2^G2A^/GFP fusion protein is restricted to the parasite cytoplasm. Proteins used as controls were predominantly found in the expected fractions consistent with the selectivity of the respective lysis procedures. In conclusion, the morphological and the biochemical analyses are consistent with the transport of *Pf*AK2/GFP to the PV and a parasite cytoplasmic localisation of *Pf*AK2^G2A^/GFP.

### The role of the putative palmitoylation site in the subcellular localization of PfAK2

**Fig 2:**
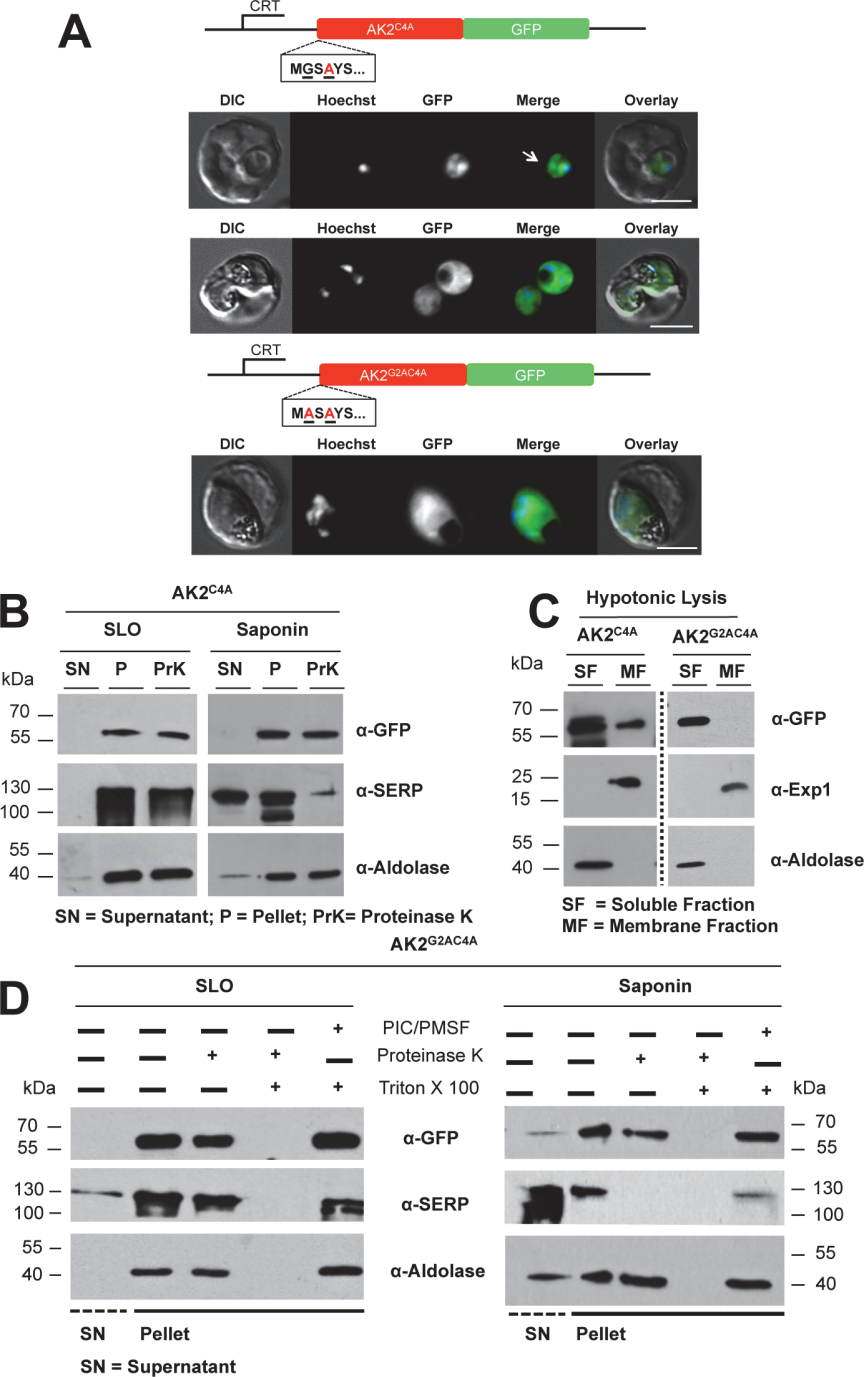
Subcellular locations of *Pf*AK2^C4A^/GFP in which the cysteine at position 4 of AK2 has been replaced with alanine, and *Pf*AK2^G2AC4A^/GFP in which the glycine (position 2) and cysteine (position 4) residues were each replaced with alanine. (A) The AK2^C4A^/GFP and AK2^G2AC4A^/GFP proteins, respectively, were expressed using the CRT promoter (construct indicated above the images). The intracellular location of the AK2^C4A^/GFP protein detected by epifluorescence microscopy in early and late trophozoite stages showed some localised patch-like structures within the parasite, a different pattern from the signal seen for *Pf*AK2/GFP or *Pf*AK2^G2A^/GFP. The intracellular location of the AK2^G2AC4A^/GFP was uniform throughout the parasite cytoplasm, and reminiscent of the distribution of *Pf*AK2^G2A^/GFP. Details as in Fig 1. (B) SLO and saponin lysis, combined with a protease protection assay for AK2^C4A^/GFP. Western blot analysis was performed with equal cell equivalents using anti-GFP, anti-SERP and anti-aldolase antibodies; labelling as in Fig 1B. No degradation of this fusion protein was detected following Proteinase K treatment of either the SLO or the saponin pellet fractions. (C) The *Pf*AK2^C4A^/GFP and *Pf*AK2^G2AC4A^/GFP transgenic parasites were subjected to hypotonic lysis, divided into soluble (SF) and membrane (MF) fractions following centrifugation, and then analysed by Western blot using anti-GFP, anti-Exp1 and anti-aldolase antibodies. (D) The pellet fractions from the SLO and saponin lysates of the AK2^G2AC4A^/GFP expressing parasites, were divided equally into four samples and either untreated, treated by addition of Proteinase K, treated by addition of Proteinase K and Triton X-100, or treated by addition of Triton X-100 and protease inhibitors (PIC/PMSF). Triton X-100 was used to dissolve the PPM so that Proteinase K was able to digest the GFP chimera once it was accessible. These data show the efficacy of protein degradation by Proteinase K (disappearance of the protein band in the corresponding fraction). Western blot analysis was performed using anti-GFP, anti-SERP and anti-aldolase antibodies. No Proteinase K degradation of the AK2^G2AC4A^/GFP fusion protein was detected after treatment of either the SLO or the saponin pellet fractions.

Apart from a glycine residue at position 2, which is indicative of *N*-myristoylation by *Pf*NMT, *Pf*AK2 has a cysteine residue at position 4 suggestive of a palmitoylation site. To study the phenotypic effect of replacing the cysteine residue at position 4, it was changed to alanine (Fig 2A). When the subcellular location of the *Pf*AK2^C4A^/GFP fusion protein was examined by fluorescence microscopy, it showed a diffuse cytosolic signal (Fig 2A). In addition, a localised but poorly defined patch-like accumulation at some regions within the parasite cytosol was seen, especially in early stages of parasite development (marked with a white arrow in Fig 2A), including the trophozoite-stage. The results obtained after differential fractionation of RBC infected with *Pf*AK2^C4A^/GFP transgenic parasites were consistent with an intra-parasite localisation; the recombinant protein was protected from protease digestion after both SLO and saponin treatment, respectively (Fig 2B).

The observation that the recombinant protein showed both a diffuse pattern and a more focused localisation prompted us to investigate its solubility characteristics. Infected RBC were subjected to hypotonic lysis and separated into a fraction containing soluble proteins and a membrane fraction. The recombinant protein was detectable in both fractions (Fig 2C). Antibodies against *Pf*Exp1—an integral membrane protein of the PVM—and *Pf*aldolase were used to detect the respective proteins. As expected, *Pf*aldolase was found almost exclusively in the fraction of soluble proteins, whereas *Pf*Exp-1 was only found in the membrane fraction. In conclusion, the *Pf*AK2^C4A^GFP variant is restricted to an intra-parasite location where it exists in both a soluble and an insoluble, possibly membrane-bound form. Thus, the substitution of the cysteine residue with an alanine residue at position 4 prevented secretion of the protein beyond the PPM. A weak membrane association of the myristoyl moiety may be the explanation for a population of the protein that does not segregate with soluble proteins.

To examine possible synergistic effects caused by modifications of both the *N*-myristoylation and putative palmitoylation sites a transgenic parasite line (*Pf*AK2^G2AC4A^/GFP parasite) was generated, which produced a recombinant GFP fusion with both the glycine and the cysteine residues exchanged for alanine residues. When examined by fluorescence microscopy, this recombinant protein showed a strong cytosolic signal, similar to the images obtained for *Pf*AK2^G2A^GFP but without the patches of focal fluorescence seen for *Pf*AK2^C4A^GFP (Fig 2A). The cytosolic localization of this GFP chimera was further supported by results obtained after hypotonic lysis. A band corresponding to the *Pf*AK2^G2AC4A^/GFP of 60 kDa could be detected only in the soluble fraction (Fig 2C). *Pf*Exp1 was detected in the membrane fraction, while *Pf*aldolase was detected in the soluble fraction showing that a clear separation took place during the lysis and centrifugation procedure (Fig 2C). Following SLO and saponin lysis of the *Pf*AK2^G2AC4A^/GFP transgenic parasite line the GFP chimera was detected in both untreated and Proteinase K-treated cell pellet fractions (Fig 2D). These results indicate that the fusion protein was not secreted beyond either the PVM or the PPM, respectively.

To investigate whether or not Proteinase K can digest the recombinant protein, the SLO and saponin cell pellet fractions were treated with Proteinase K in the presence of a detergent, Triton X-100, which permeabilises the PPM allowing Proteinase K access to the parasite cytosol. Whilst the *Pf*AK2^G2AC4A^/GFP band was present following treatment of the parasitized cells with Triton X-100, in the presence of Triton X-100 and Proteinase K the band was not present, indicating that it had been degraded by this protease (Fig 2D).

From these data it can be concluded, that the *Pf*AK2^G2AC4A^/GFP protein is located in the parasite cytosol according to both fluorescence and biochemical analyses.

### The role of the cluster of basic residues in the subcellular localization of PfAK2

**Fig 3:**
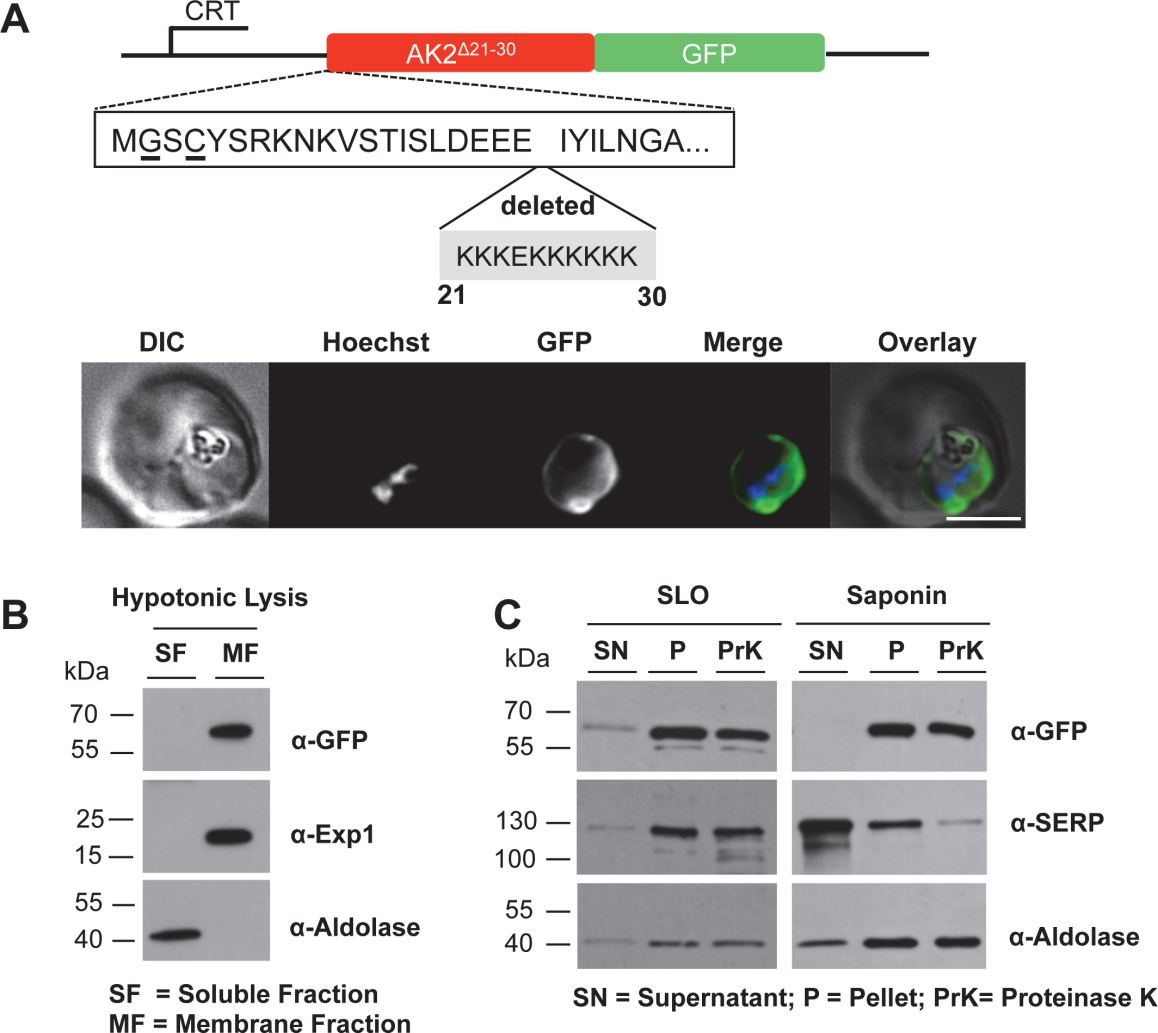
Deletion of the stretch of basic residues at the N-terminus of *Pf*AK2 alters the subcellular location of the protein. (A) The AK2^Δ21-30^/GFP was expressed using the CRT promoter (construct indicated above the images). Live cell imaging of the *Pf*AK2^Δ21-30^/GFP parasite line in a late trophozoite stage parasite; details as in Fig 1. The AK2^Δ21-30^/GFP transgenic parasites were subjected to (B) hypotonic, or (C) SLO and saponin lysis and cell fractionation as described in the legend to Fig 2. No degradation of the fusion protein was detected after Proteinase K treatment of either the SLO or the saponin pellet fractions. The labelling is as in Figs 1 and 2.

In addition to the putative palmitoylation and myristoylation sites, PfAK2 contains a third conspicuous element, namely a stretch of lysine residues at positions 21 to 23 and 25 to 30. To investigate the role of this block of basic residues (nine lysines surrounding one acidic glutamate residue) the codons were deleted via overlapping extension PCR and the resultant construct *Pf*AK2^Δ21-30^/GFP was transfected into parasites. Live cell imaging showed a peripheral staining with a protrusion similar to that obtained with the wild type AK2/GFP chimera (Fig 3A). Hypotonic lysis of infected erythrocytes followed by centrifugation resulted in an almost quantitative sedimentation of the GFP-chimera in the membrane fraction (Fig 3B). In both the SLO and saponin treated samples, a band corresponding to the size of the GFP chimera was detected in the cell pellet fractions, which was not susceptible to Proteinase K (Fig 3C). The morphological analysis and the data obtained by differential cell lysis in combination with protease treatment are consistent with an intra-parasite location of *Pf*AK2^Δ21-30^/GFP protein, presumably at the cytoplasmic face of the parasite plasma membrane.

### The N-terminus of PfAK2 is sufficient for secretion of GFP

**Fig 4:**
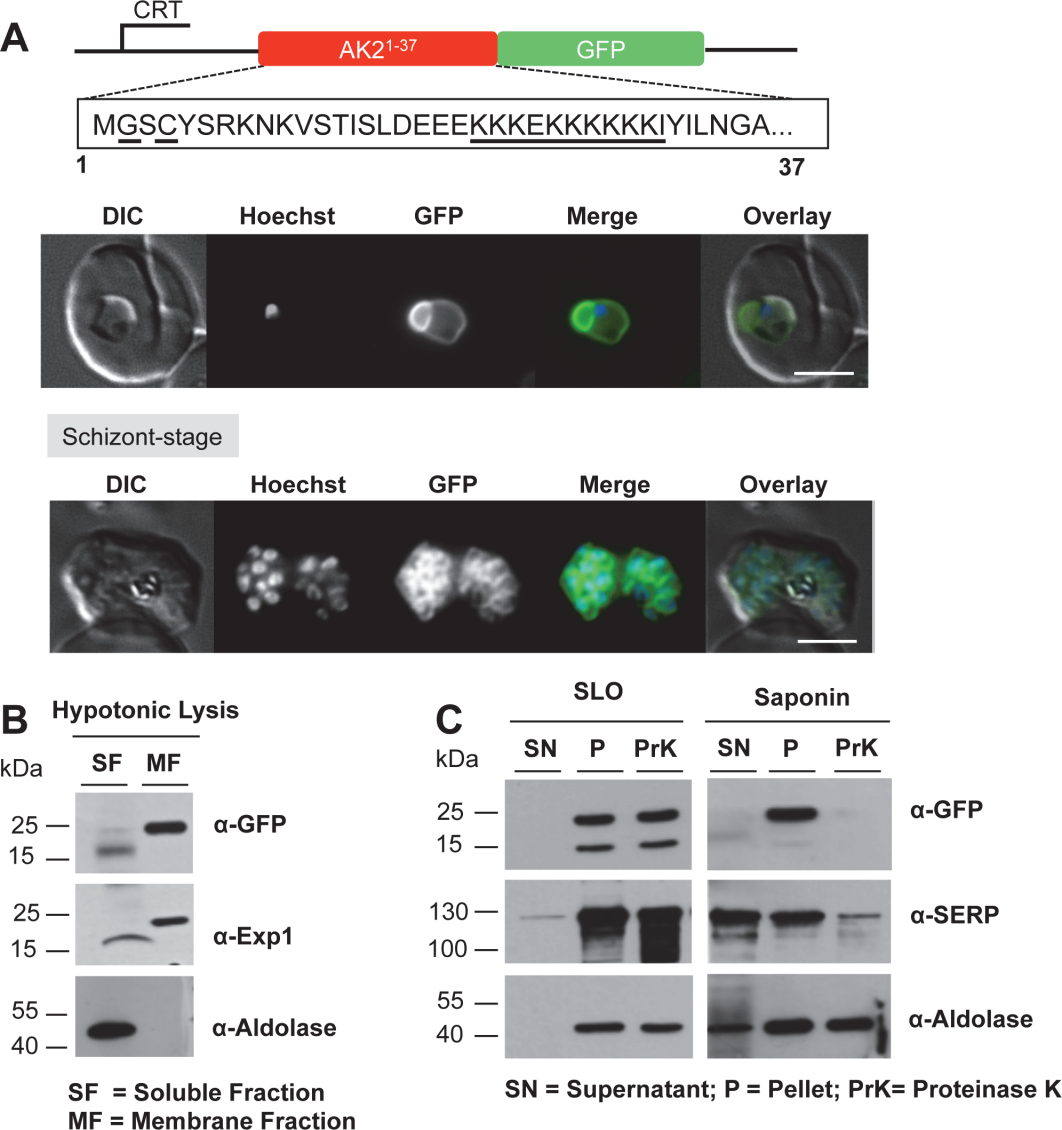
The *Pf*AK2 N-terminus targets GFP to the outside of the parasite plasma membrane. (A) The AK2^1-37^/GFP was expressed using the CRT promoter (construct indicated above the images). Live cell imaging of the *Pf*AK2^1-37^/GFP in trophozoite and schizont stages, showed a location of the GFP signal similar to that of *Pf*AK2/GFP. In the schizont stage of these parasites a clear signal for the fusion protein was visible around each of the individual daughter merozoites. Details as in Fig 1. The AK2^1-37^/GFP transgenic parasites were subjected to (B) hypotonic, or (C) SLO and saponin lysis and cell fractionation as described in Fig 2. The absence of the protein band corresponding to the *Pf*AK2^1-37^/GFP fusion protein in the saponin pellet fraction treated with Proteinase K indicates the secretion of the protein beyond the PPM. The labelling is as in Figs 1 and 2.

The preceding mutagenesis analyses of *Pf*AK2 revealed that the *N*-myristoylation site is required for membrane binding and that the cysteine at residue 4 likely plays a role in stabilizing the membrane anchoring of this protein, presumably through palmitoylation. However, secretion of *Pf*AK2 beyond the PPM was not achieved when a cluster of basic amino acids was removed from the protein, indicating a potential role for this lysine-rich structure in membrane translocation. Therefore, to determine whether or not the N-terminus of *Pf*AK2 including these three motifs was sufficient to mediate secretion beyond the PPM, a chimeric construct was generated to produce the first 37 amino acids of the *Pf*AK2 protein sequence containing all three motifs fused to GFP (Fig 4A), and transfected into the parasite. Analysis of the transgenic parasites revealed a similar pattern of fluorescence to that of parasites transfected with *Pf*AK2/GFP; that is a ring-like structure, indicating peripheral staining with a clear outward protrusion (Fig 4A). Hypotonic lysis of infected erythrocytes followed by centrifugation localized the GFP-chimera exclusively to the membrane fraction (Fig 4B). In both the SLO and saponin treated samples a band corresponding to the size of the GFP chimera was detected in the cell pellet fractions, but in the presence of Proteinase K the fusion protein was absent from the saponin cell pellet fraction, indicating that it was accessible to the protease outside the PPM (Fig 4C).

To discriminate between a location at the external face of the PPM and a location at the vacuolar face of the PVM we examined segmented schizonts that contain developing merozoites prior to their release from the infected RBC and still surrounded by the PVM. In RBC containing clearly distinguishable merozoites, GFP fluorescence was restricted to the surface of individual developing merozoites and absent from the PVM (Fig 4A, schizont stage). For these late stage parasites, the solubility characteristics and the accessibility to Proteinase K of the fusion protein were similar to those of the protein in trophozoites (data not shown), and therefore, it is likely that at both stages the fusion protein is associated with the outer face of the PPM.

### A modified N-terminus of PfARF1 localizes GFP to the parasite plasma membrane but does not lead to subsequent translocation across the PPM

**Fig 5:**
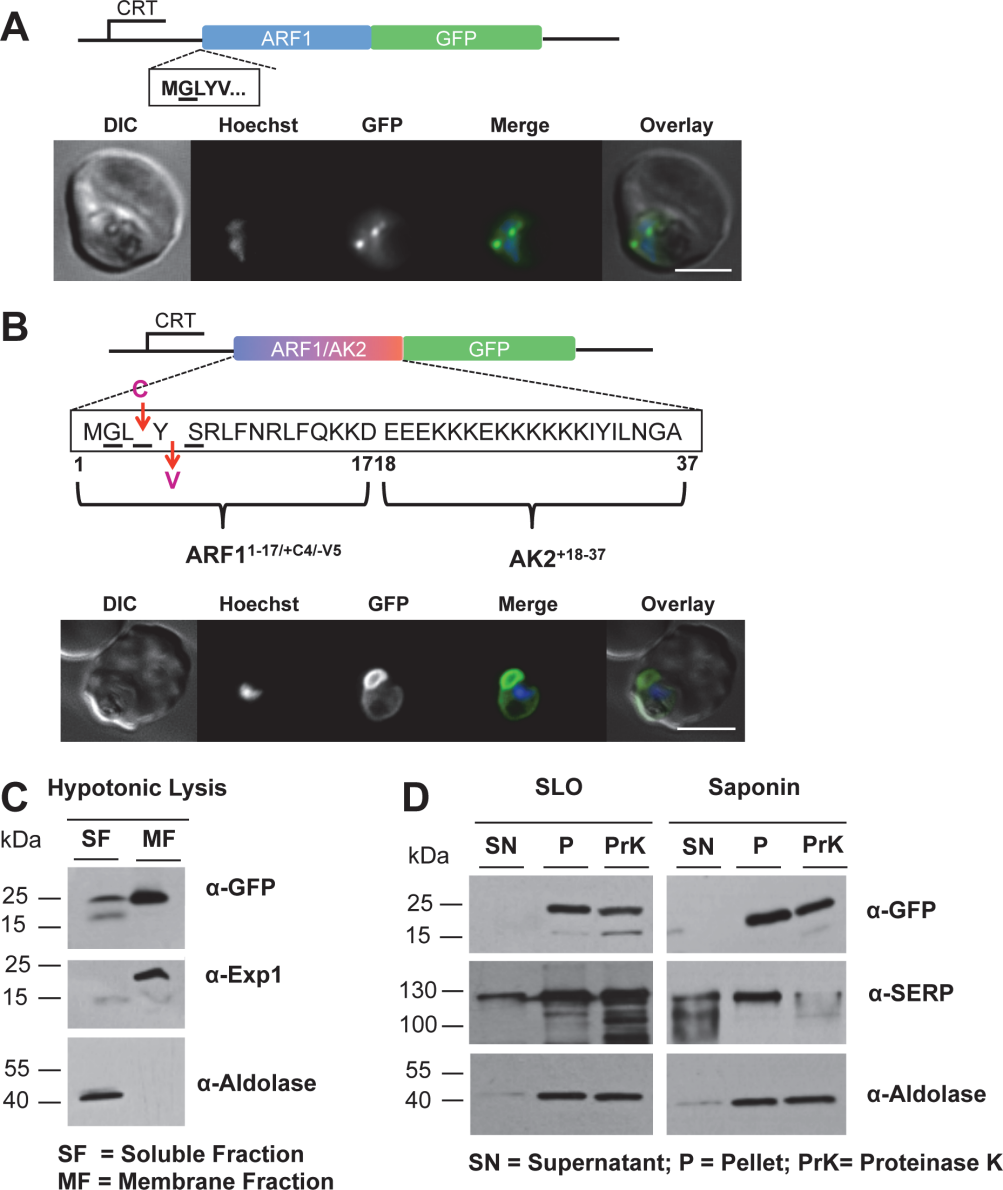
The subcellular location of *Pf*ARF1/GFP and GFP fused to the modified version of the N-terminus of ARF1, *Pf*ARF^1-17/+4/-5^AK2^18-37^/GFP. (A) *Pf*ARF1/GFP was expressed using the CRT promoter (construct indicated above the images) and detected by epifluorescence microscopy. *Pf*ARF1/GFP was largely located in discrete dots; Figure details as in Fig 1. (B) The chimeric *Pf*ARF^1-17/+4/-5^AK2^18-37^/GFP was expressed using the CRT promoter (construct indicated above the images). Live cell imaging of the *Pf*ARF^1-17/+4/-5^AK2^18-37^/GFP parasite line at the late trophozoite stage showed that the pattern of GFP expression was similar to that of *Pf*AK2/GFP. Figure details are as in Fig 1. The *Pf*ARF^1-17/+4/-5^AK2^18-37^/GFP transgenic parasite was subjected to (C) hypotonic, or (D) SLO and saponin lysis and cell fractionation as described in Fig 2. No degradation of the fusion protein was detected after Proteinase K treatment of either the SLO or the saponin pellet fractions, indicating that the protein is protected from the protease and therefore within the PPM. The labelling is as in Figs 1 and 2.

To further validate the results obtained with *Pf*AK2 we studied a second protein, *Pf*ARF1. *Pf*ARF1 is a protein highly conserved across eukaryotes, involved in vesicular shuttling in other organisms [19] and known to be myristoylated in the parasite [13]. A plasmid was constructed for the expression of *Pf*ARF1 fused to GFP and transfected into the *P*. *falciparum* 3D7 strain for episomal expression. By fluorescence microscopy, the *Pf*ARF1/GFP was found localized to discrete dot-like structures in the trophozoite/schizont cytosol (Fig 5A). Following hypotonic lysis and separation into soluble and membrane fractions by centrifugation, western blot analysis with antibodies to GFP detected a band of approximately 50 kDa in both the soluble supernatant and membrane pellet fractions of the *Pf*ARF1/GFP parasite, corresponding to the expected size of the fusion protein (full-length *Pf*ARF1 is 22 kDa and GFP is 27 kDa) (S1A Fig). These results indicate that the myristoylated ARF1/GFP was partially, but not exclusively, membrane bound, presumably mediated by the N-terminal myristate moiety. Further analysis using Grasp1 [11], a verified marker protein of the Golgi compartment, showed that *Pf*ARF1/GFP and Grasp1/mCherry co-localized, presumably at the Golgi complex (S1B Fig).

In contrast to *Pf*AK2, *Pf*ARF1 lacks the putative palmitoylation site and cluster of basic amino acids close to the N-terminus. To determine whether these two motifs together with N-myristoylation were sufficient for trafficking ARF1 to the PV, a chimeric construct was generated consisting in part of sequence coding for the N-terminus of ARF1 and in part for the N-terminus of AK2 fused to GFP-coding sequence in the pARL2 vector, prior to transfection into *P*. *falciparum* (Fig 5B). The protein comprised the first 17 residues of ARF in which a cysteine had been inserted at position 4 and a valine deleted at position 5, fused with AK2 residues 18 to 37 and GFP (*Pf*ARF^1-17/+4/-5^AK2^18-37^/GFP). Fluorescence analyses showed a GFP pattern similar to that of the *Pf*AK2/GFP with a peripheral distribution and a protrusion (Fig 5B). Biochemical analyses showed a strong GFP signal corresponding to the GFP chimera in the membrane fraction after hypotonic lysis and cell fractionation, with a faint band also in the soluble fraction (Fig 5C). A strong GFP signal was also detected in both the SLO and saponin cell pellet fractions, in either the presence or absence of Proteinase K, indicating that the *Pf*ARF^1-17/+4/-5^AK2^18-37^/GFP fusion protein was within the parasite and protected by the PPM from digestion (Fig 5D). These results indicate that the myristoylation and putative palmitoylation sites together with the lysine rich region are not sufficient to provide translocation across the PPM and therefore there is additional information in the first 37 amino acids of AK2 that is essential for this translocation. Alternatively, sequences contained in this construct prevent membrane translocation.

## Discussion

In this study we propose unconventional mechanisms of protein trafficking to the PVM and beyond have been proposed, and signals have been identified. We identify putative topogenic signals leading to secretion of the *Pf*AK2 protein to the outer face of the PPM. Three elements were investigated, the N-terminal myristoylation, a cysteine that is a putative palmitoylation site, and a stretch of basic amino acids, all within the N-terminal 37 amino acid residues of *Pf*AK2. *Pf*AK2 and variants thereof were produced as fusion proteins with GFP to allow the location of the protein in the parasite-infected RBC to be investigated by fluorescence microscopy, and following biochemical fractionation of the cell to identify the corresponding subcellular compartment. By using SLO and saponin to selectively lyse the RBC PM, and the RBC PM and PVM, respectively, together with addition of Proteinase K to define accessibility to added protease, it was possible to define three distinct locations: inside the parasite, in the PV lumen and outside the PV. Hypotonic cell lysis and resolution of the products by centrifugation to yield soluble and membrane fractions enabled the membrane partitioning of the proteins to be investigated.

*Pf*AK2 lacks a signal peptide, but contains a *N*-myristoylation site and was proposed to be secreted beyond the PPM to the PVM [18], based on fluorescence imaging and co-localization with *Pf*Exp1. Ma and colleagues had also shown that the subcellular location of *Pf*AK2 differed depending on the presence or absence of the *Pf*AK2 *N*-myristoylation site [18]. We extended these earlier studies by combining live fluorescence imaging with biochemical approaches. We found that *Pf*AK2/GFP was located at the periphery of the parasite and secreted beyond the PPM, probably within the PV, attached to the outer leaflet of the PPM.

In a next step we examined the importance of other sequence features at the N-terminus of *Pf*AK2, focusing on the putative palmitoylation site and the stretch of basic residues. Although other myristoylated proteins may also contain either a cluster of basic residues or are dually acylated for stable membrane association, the *Pf*AK2 protein contains all three of these elements. Möskes and colleagues suggested that these three motifs were required to drive CDPK1 into the PV [10], although their study had no biochemical data such as protection from proteinase digestion to support the proposed location.

Removal of the putative palmitoylation site as in the *Pf*AK2^C4A^/GFP variant resulted in a protein not secreted beyond the PPM. The lack of a putative palmitoylation site close to the N-terminus of the protein also led to only a fraction of the protein being associated with membrane, possibly due to a reversible weak interaction between the *N*-myristoyl group and parasite membrane. This suggestion is in accordance with the proposed 'two-step model' for the membrane binding of many dual acylated proteins including members of the Src family [22]. Furthermore, the AK2^C4A^/GFP variant showed a patch-like accumulation and an uneven distribution in the parasite cytosol, suggesting that the protein partly associates with intracellular membranes. A similar phenotype was observed in the studies of *Pf*CDPK1^C3A^/GFP (putative palmitoylation site removed) [10] and of a palmitoylation-deficient variant of the *Leishmania* HASPB [16]. Denny and colleagues observed some association of the palmitoylation-deficient HASPB with the outer leaflet of the Golgi, and they proposed a model where HASPB is normally co-translationally myristoylated in the cytoplasm, then trafficked to the Golgi membrane to be palmitoylated by a palmitoyltransferase at the outer leaflet of the Golgi before being targeted and translocated across the plasma membrane [16].

The third motif—the cluster of lysine residues—in close proximity to the dual acylation sites—seems to play a role in membrane attachment for other proteins, possibly due to electrostatic interactions with acidic phospholipids of the PPM [23]. It is found in many myristoylated proteins including Src and MARCKS and plays a role in membrane targeting and binding [22], although it cannot be ruled that it merely has a spacer function in AK2. Deletion of the row of lysine residues, as performed with the *Pf*AK2^Δ21-30^/GFP variant, resulted in a protein not secreted beyond the PPM.

We showed next that the N-terminal 37 amino acids of *Pf*AK2 were sufficient to direct GFP for translocation across the PPM. These results mirror those obtained with the *Leishmania* HASPB—for which the N-terminal 18 amino acids were sufficient to target the protein to the cell surface. In addition, replacement of the respective acylation sites led to a retention of this protein in the parasite cytosol [16], similar to our findings with *Pf*AK2.

To determine whether or not these three motifs are sufficient to drive secretion of another *N*-myristoylated protein, we first established the properties of *Pf*ARF1 [9, 13]. The full-length fusion protein *Pf*ARF1/GFP localized to the cytosol of the parasite as one or two dot-like structures with soluble and membrane-bound fractions within the trophozoite. *Pf*ARF1/GFP was likely targeted to the Golgi apparatus of the secretory pathway, showing strong co-localization with the Golgi marker, Grasp 1 and in accordance with the findings for ARF1 in human cells, which is mostly present at the *cis*-Golgi complex [24] and involved in vesicle formation and trafficking [25]. We then generated and analysed a chimeric sequence between the N-termini of *Pf*ARF1 and *Pf*AK2. However, the incorporation of a putative palmitoylation site and the addition of a row of basic residues in close proximity to the *N*-myristoylation site of *Pf*ARF1 only targeted the GFP chimera to the cytosolic side of the PPM but were not sufficient to translocate this protein beyond the PPM. This suggests that the information for the translocation of AK2 found in the first 37 amino acids includes other features, including conformational constraints, in addition to the dual acylation and row of basic residues.

Overall, the results obtained for *Pf*AK2 indicate a secretory mechanism different from trafficking through the ER/Golgi route, and similar to that found for *Leishmania* HASPB [16] and proposed for *Pf*CDPK1 [10]. The existence of such alternative pathways has already been described for a small number of proteins in various eukaryotic systems [15] and the number of known proteins using them is steadily growing. In the *P*. *falciparum* genome there are a number of genes coding for proteins with a predicted *N*-myristoylation site, a putative palmitoylation site and a large number of basic amino acids in the N-terminal 50 amino acids. These proteins may also be translocated across the PPM in a similar way, and include a calpain (PF3D7_1362400) [26], a protein phosphatase (PF3D7_0810300) [27], and CDPK1 (PF3D7_0217500) [10]. It would be of interest to analyse these candidates using the methods described here to see whether or not they are also exported in the same way as *Pf*AK2. Published data suggest that of these three proteins only CDPK1 may be exported in a similar way

An interesting observation revealed in this study using the *Pf*AK2/GFP fusion protein and some of the variants is the presence of the protuberances, representing extensions of the PPM apparently extending away from the parasite into the host cell cytoplasm. It has already been proposed that these structures are membranous [18], and similar structures have been observed by immunofluorescence of liver stage parasites [28]. However the identity and composition of these protrusions is not known. It would be of interest to establish whether they contained specific proteins and/or an accumulation of certain lipids.

*Pf*NMT has been validated as a promising drug target since several essential parasite proteins require *N*-myristoylation to perform their biological function [13]. For example inhibition of NMT prevents formation of the inner membrane complex, nuclear division, and further parasite development [13]. The discovery and characterization of a broader range of substrates and the role of *N*-myristoyltransferase in different cellular processes contributes to the understanding of the importance of this protein modification in membrane targeting and also protein secretion.

## Materials and Methods

### Cultivation of parasites

The *P*. *falciparum* 3D7 strain was cultured as described [29] in human red blood cells obtained as donations from anonymised individuals from the Bloodbank, UniversitätsKlinikum Giessen und Marburg (http://www.ukgm.de). Late-stage parasites were synchronized using Gelafundin (a gelatin derivative) flotation [30] and a high gradient magnetic field [31]. The parasites were synchronized by sorbitol treatment at the ring-stage [32], prior to transfection using the protocol described previously [33].

### Plasmid construction via polymerase chain reaction and site-directed mutagenesis

The vector pARL2GFP contains the β-lactamase and human DHFR selectable markers, providing resistance to ampicillin and WR99210, respectively, and was used to produce proteins with a GFP tag at the C-terminus expressed from the *crt* promoter [34]. pARL2_mCherry_BSD contains the β-lactamase and blasticidin-S-deaminase selectable markers, providing resistance to ampicillin and blasticidin, respectively, and was used to produce protein with a mCherry tag at the C-terminus and expressed from the *crt* promoter. The coding sequences of *Pf*AK2 and the variant (AK2^G2A^) were cloned into the pARL2-GFP vector [34]. Further plasmids to produce additional *Pf*AK2 variants were designed in which the codons for various amino acid sequences at the N-terminus of the protein were replaced or deleted. Primers are listed in S1 Table. The coding region of AK2 was amplified with KOD polymerase (Novagen) using the AK2^C4A^_XhoI_F forward primer (to replace the putative palmitoylation site, Cys^4^ with alanine) and the AK2_AvrII_R reverse primer, digested with XhoI and AvrII (NEB) and cloned into XhoI/AvrII digested pARL2-GFP vector [34]. A double-mutant was created by changing the codons for the *N*-myristoylation site, Gly^2^, and the putative palmitoylation site, Cys^4^, to alanine codons using the AK2^G2AC4A^ forward primer. The sequence coding for the polybasic-cluster at the N-terminus of *Pf*AK2 (KKKEKKKKKK) was deleted in two PCR reaction steps [35] using amplification with two internal primers (S1 Table) in addition to the flanking AK2_XhoI_F and AK2_AvrII_R primers, digestion with XhoI and AvrII and cloning into XhoI/AvrII digested pARL2-GFP plasmid. Sequence coding for the N-terminal 37 amino acids of *Pf*AK2 was amplified with the AK2_XhoI_F and the AK2_KpnI_R primers, digested with XhoI and KpnI and fused upstream to GFP sequence in the XhoI/KpnI digested pARL2-GFP vector.

Amplification of the coding sequence of *Pf*ARF1 was performed using the SuperScript III one-step RT-PCR kit (Invitrogen) with the primers ARF1_XhoI_F and ARF1_AvrII_R and total RNA isolated from *P*. *falciparum*. After digestion with XhoI and AvrII, the DNA was ligated into XhoI/AvrII digested pARL2-GFP.

A chimeric construct of *Pf*ARF1 and *Pf*AK2 sequences was generated to add a putative palmitoylation site, Cys^4^ at the 4th position of the N-terminus of ARF1 and to fuse the first 17 amino acids of ARF1 to the AK2 sequence containing the cluster of lysine residues (amino acids 18–37). This coding sequence was generated by Geneart synthesis (Invitrogen), digested with XhoI and KpnI restriction enzymes and ligated into the XhoI/KpnI digested pARL2-GFP vector. The sequence of each of the plasmids was verified by Seqlab (Göttingen) or GATC (Konstanz).

### Cell fractionation of infected erythrocytes

Trophozoite-infected cells were enriched using Gelafundin flotation or magnet based separation and subjected to hypotonic lysis. A total of 2 x 10^8^ cells was resuspended in 1 mM Tris and lysed by repeated cycles of freezing in liquid nitrogen and thawing. Following centrifugation at 36,000 *g* for 20 minutes (min) at 4°C, the lysate was separated into soluble and pellet fractions. The soluble fraction was centrifuged again at the same speed to remove any remaining membrane contaminants. The pellet fraction was washed 4–6 times in PBS (pH 7.4) containing 1 mM PMSF and protease inhibitor cocktail (PIC; Calbiochem; 1:200 dilution). Both fractions were mixed with sample buffer, placed at 100°C for 10 min and then analysed by SDS-PAGE and immunoblotting.

### Streptolysin O (SLO) lysis of parasitized cells

Immediately after enrichment of the trophozoite-stage infected erythrocytes a total of 2 x 10^8^ cells was treated with 3 hemolytic units (HU) of SLO (kindly provided by Professor S. Bhakdi, University of Mainz) in 188 μl of PBS (pH 7.4) [36]. The cells were incubated at room temperature for 6 min with gentle mixing every 2 minutes. Following incubation the cells were spun at 1,000 *g* for 3 min. The supernatant was transferred to a new 1.5 ml Eppendorf tube, and centrifuged two more times to remove any remaining contamination with the cellular and membrane fraction. The pellet was washed 4 to 6 times with PBS (pH 7.4) containing 1 mM PMSF (AppliChem) and PIC (1:200 dilution). Subsequently, the pellet containing the intact PV and parasites was resuspended in an appropriate volume of PBS (pH 7.4). Fractions were mixed with sample buffer and immediately heated at 100°C for 10 min before being used for experiments.

### Saponin lysis of parasitized cells

A total of 2 x 10^8^ infected erythrocytes enriched with trophozoite-stage parasites was treated with 0.02% (w/v) saponin (Roth; stock: 1mg/ml) in PBS (pH 7.4) containing 1 mM PMSF [37]. The cells were incubated at room temperature for 3 min with gently mixing, and then they were immediately centrifuged at 2,800 *g* for 5 min at 4°C. The supernatant was transferred to a new 1.5 ml Eppendorf tube, whilst the pellet was washed 4–6 times with PBS (pH 7.4) containing 1 mM PMSF and PIC (1:200 dilution). To remove all the residual membrane from the supernatant fraction another centrifugation step was performed at 36,000 *g* for 20 min at 4°C. The pellet was suspended in PBS (pH 7.4) containing 1 mM PMSF and PIC (1:200 dilution). SDS-PAGE sample buffer was added to both fractions and then they were heated at 100°C for 10 min before further analysis.

### Protease protection assay

The protease protection assay was performed with trophozoite-stage parasites that had been treated with 0.02% saponin or SLO (3 HU), respectively, as described above. The supernatant and pellet fractions were prepared by centrifugation at 2,800 *g* for 5 min and the saponin or SLO pellet was washed 4–6 times with PBS (pH 7.4) and divided for the majority of the experiments into two samples each containing 1 x 10^8^ cell equivalents. Sample 1 was resuspended in PBS (pH 7.4) and not treated, as the negative control, whilst sample 2 was treated with 1 mg/ml Proteinase K (AppliChem). Following incubation on ice for 30 min, 1 mM PMSF and PIC were added to the samples for 3 min at room temperature to prevent further protease activity. SDS-PAGE sample buffer was added to the samples and they were heated at 100°C for 10 min prior to analysis or storage at −80°C.

### Protein analysis

All cell fractions were analysed by SDS-PAGE and western blotting using the following primary antibodies: anti-SERP (1:500 dilution), anti-aldolase (1:5000), anti-Exp1 (1:500), anti-Band 3 (1:1000; Sigma Aldrich), and anti-GFP (1:1000; Roche). The secondary antibodies were HRP-conjugated anti-mouse or anti-rabbit IgG (1:2000 dilution; DAKO, Santa Cruz), as appropriate.

### Live cell imaging

*P*. *falciparum*-infected RBC were visualized using the Zeiss Axio Observer inverted epifluorescence microscope system with the appropriate filter sets and using Axiovision 4 software. The cells were harvested by centrifugation and washed 3 times with RPMI 1640 medium. For DNA staining 10 μg/ml Hoechst 33258 dye was added to the cells, which had been resuspended in 1 ml RPMI 1640, and incubated for 5 to 10 min at room temperature on a shaker. The cells were then applied directly to a glass slide and observed at room temperature, using appropriate exposure times to prevent photo-bleaching.

Images acquired with the epifluorescence microscope were imported into ImageJ64, converted to 8-bit greyscale and subjected to background subtraction. Using the ImageJ plugin RGBmerge the RGB channels were split into red, green and blue before the individual images were overlaid. In order to make the final figures, the images were imported from ImageJ into PowerPoint (Microsoft), compiled and saved as TIF files. Before the images were saved as TIF files they were adjusted with the brightness/contrast tool. All the data shown are representative of at least 6 to12 images acquired for each sample.

## Supporting Information

S1 Fig *Pf*ARF1/GFP is partially membrane-bound and partially soluble and localizes to the Golgi compartment of the secretory pathway. (A) Cell fractionation of the *Pf*ARF1/GFP sample. Infected cells were separated into soluble (SF) and membrane (MF) fractions and subjected to Western blot analysis using anti-GFP, anti-Band 3 and anti-aldolase antibodies. Size markers in kDa are shown. (B) The ARF1/GFP and the Golgi marker Grasp1/mCherry were each expressed using the CRT promoter (construct of Grasp1 with mCherry indicated above the images). Live cell imaging of the parasites showed dot-like structures for the *Pf*Grasp1/mCherry—a marker of the Golgi-complex [11]—within the parasite, which strongly overlapped with the dot-like structures of *Pf*ARF1/GFP. The infected cell was visualised by differential interference contrast (DIC), and parasite nuclei were detected by Hoechst staining. The intrinsic fluorescence of the GFP and mCherry identified the location of the AK2/GFP fusion protein and the Grasp1/mCherry fusion protein, respectively. In the merge: green (GFP), red (mCherry), blue (Hoechst; DNA). Scale bar—3 μm.
(TIF)

S1 Table *O*ligonucleotides used in this study. (DOCX)
