# Supplementary figures and images for: Alternative Protein Secretion in the Malaria Parasite Plasmodium falciparum
Source: PLoS One. 2015 Apr 24;10(4):e0125191. doi: 10.1371/journal.pone.0125191 (PMC4409355; doi:10.1371/journal.pone.0125191)

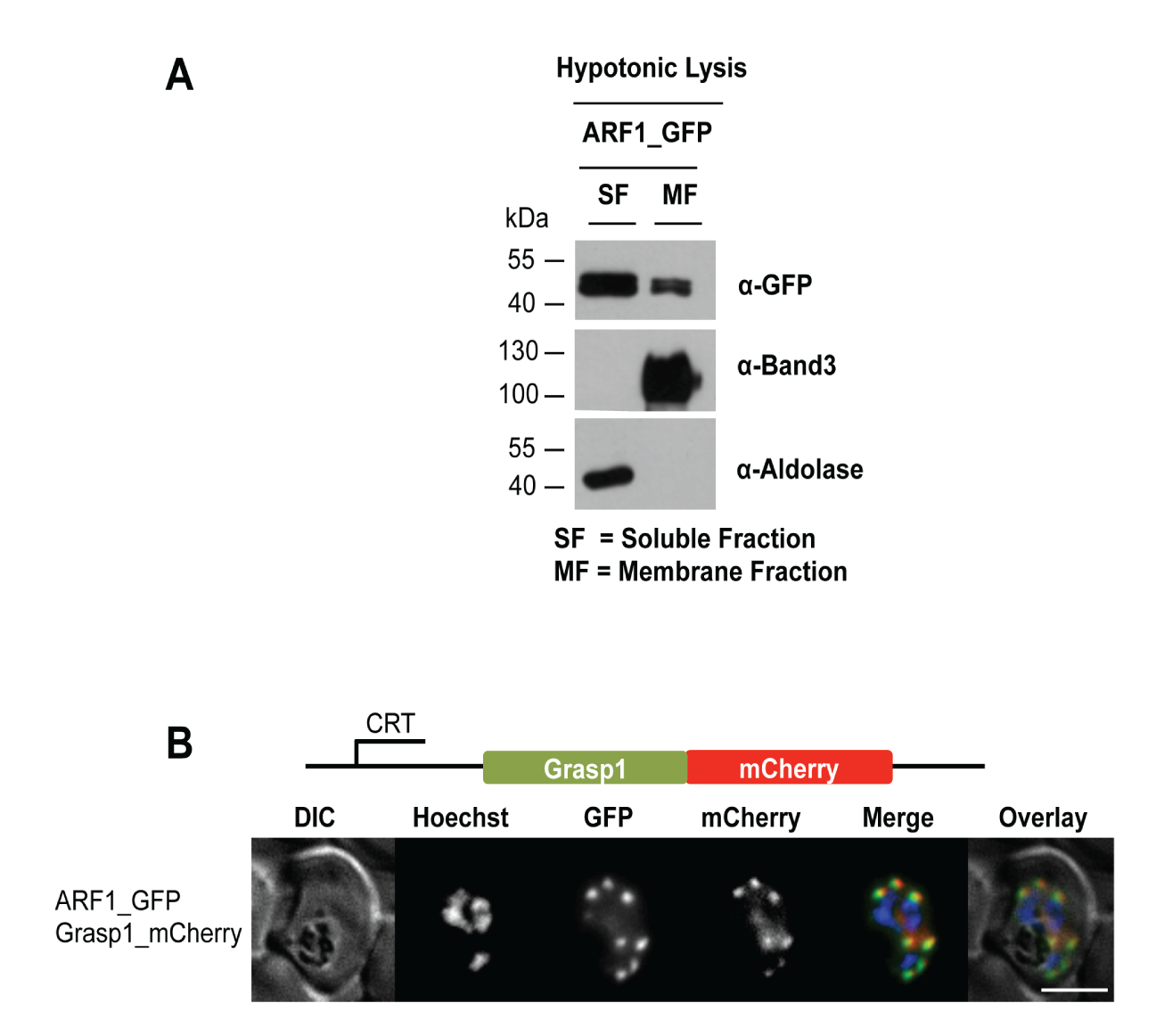

Supplement: S1 Fig — (A) Cell fractionation of the PfARF1/GFP sample. Infected cells were separated into soluble (SF) and membrane (MF) fractions and subjected to Western blot analysis using anti-GFP, anti-Band 3 and anti-aldolase antibodies. Size markers in kDa are shown. (B) The ARF1/GFP and the Golgi marker Grasp1/mCherry were each expressed using the CRT promoter (construct of Grasp1 with mCherry indicated above the images). Live cell imaging of the parasites showed dot-like structures for the PfGrasp1/mCherry—a marker of the Golgi-complex [11]—within the parasite, which strongly overlapped with the dot-like structures of PfARF1/GFP. The infected cell was visualised by differential interference contrast (DIC), and parasite nuclei were detected by Hoechst staining. The intrinsic fluorescence of the GFP and mCherry identified the location of the AK2/GFP fusion protein and the Grasp1/mCherry fusion protein, respectively. In the merge: green (GFP), red (mCherry), blue (Hoechst; DNA). Scale bar—3 μm. (TIF) [file pone.0125191.s001.tif]
