# Supplementary material for: Alternative Protein Secretion in the Malaria Parasite Plasmodium falciparum
Source: PLoS One. 2015 Apr 24;10(4):e0125191. doi: 10.1371/journal.pone.0125191 (PMC4409355; doi:10.1371/journal.pone.0125191)
Supplement: S1 Table — (DOCX) [file pone.0125191.s002.docx]

**S1_Table**. ***O*ligonucleotides used in this study**. Restriction enzyme sites are underlined and mutations in codons for introduction by site-directed mutagenesis are highlighted in bold.

| **Name** | **Nucleotide sequence** |
| --- | --- |
| ***P. falciparum* adenylate kinase 2 (PF3D7_0816900)** | |
| AK2_XhoI_F | GGCTCGAGATGGGATCATGTTATAGTAGAAAAAATAAA |
| AK2^G2A^_XhoI_F | GGCTCGAGATG**GCA**TCATGTTATAGTAGAAAAAATAAA |
| AK2^C4A^_XhoI_F | GGCTCGAGATGGGATCA**GCT**TATAGTAGAAAAAATAAA |
| AK2^G2AC4A^_XhoI_F | GGCTCGAGATG**GCA**TCA**GCT**TATAGTAGAAAAAATAAA |
| AK2_AvrII_R | GGCCTAGGATTGGGGTTATCATCTATAATGGAG |
| AK2^(Δ21-30)^_F_oe-PCR* | CATTAGATGAAGAGGAAATATATATTTTAAATGGAGCATCTGGG |
| AK2^(Δ21-30)^_R_oe-PCR* | GATGCTCCATTTAAAATATATATTTCCTCTTCATCTAATGATATTGTTG |
| AK2^1-37^_KpnI_R | GGGGTACCTGCTCCATTTAAAATATATATTTTTTTTTTCTTCTT |
| ***P. falciparum* ADP-ribosylation factor 1 (PF3D7_1020900)** | |
| ARF1_XhoI_F | GGCTCGAGATGGGTTTATATGTAAGTAGGTTATTTAATCG |
| ARF1_AvrII_R | GGCCTAGGTTTGGCATTATTTAAGTGTGTGGTTAGC |
| ARF1^1-17/+ C4/-V5/^AK2^+18-37^ _XhoI (F) _ KpnI (R) | **GG**CTCGAGATG**GGT**TTA**TGC**TATAGTAGGTTATTTAATCGTTTATTTCAAAAGAAA**GATGAAGAGGAAAAAAAGAAGAGAAAAAAAAGAAAAAGAAAATATATATTTTAAATGGAGCA**GGTACC**GC** |
| ***P. falciparum* Golgi reassembly stacking protein1 (PF3D7_101700.1)** | |
| Grasp1_XhoI_F | GGCTCGAGATGGGAGCAGGACAAACG |
| Grasp1_AvrII_R | GGCCTAGGCAATATGTTCTTTCTTAC |

* primers for overlapping-extension PCR (oe-PCR)
